# Flexible Platform of Acoustofluidics and Metamaterials with Decoupled Resonant Frequencies

**DOI:** 10.3390/s22124344

**Published:** 2022-06-08

**Authors:** Shahrzad Zahertar, Hamdi Torun, Chao Sun, Christopher Markwell, Yinhua Dong, Xin Yang, Yongqing Fu

**Affiliations:** 1 Faculty of Engineering and Environment, Northumbria University, Newcastle upon Tyne NE1 8ST, UK; s.zahertar@soton.ac.uk (S.Z.); christopher.markwell@northumbria.ac.uk (C.M.); 2 Zepler Institute, University of Southampton, Southampton SO17 1BJ, UK; 3 School of Life Sciences, Northwestern Polytechnical University, Xi’an 710072, China; chaosun@nwpu.edu.cn; 4 Department of Neurology, Tianjin 4th Centre Hospital Affiliated to Nankai University, Tianjin 300140, China; yinhuadong@nankai.edu.cn; 5 Department of Electrical and Electronic Engineering, School of Engineering, Cardiff University, Cardiff CF24 3AA, UK; yangx26@cardiff.ac.uk

**Keywords:** electromagnetic metamaterials, acoustofluidics, surface acoustic waves, microfluidics, hybrid physical and chemical sensors, droplet actuation

## Abstract

The key challenge for a lab-on-chip (LOC) device is the seamless integration of key elements of biosensing and actuation (e.g., biosampling or microfluidics), which are conventionally realised using different technologies. In this paper, we report a convenient and efficient LOC platform fabricated using an electrode patterned flexible printed circuit board (FPCB) pressed onto a piezoelectric film coated substrate, which can implement multiple functions of both acoustofluidics using surface acoustic waves (SAWs) and sensing functions using electromagnetic metamaterials, based on the same electrode on the FPCB. We explored the actuation capability of the integrated structure by pumping a sessile droplet using SAWs in the radio frequency range. We then investigated the hybrid sensing capability (including both physical and chemical ones) of the structure employing the concept of electromagnetic split-ring resonators (SRRs) in the microwave frequency range. The originality of this sensing work is based on the premise that the proposed structure contains three completely decoupled resonant frequencies for sensing applications and each resonance has been used as a separate physical or a chemical sensor. This feature compliments the acoustofluidic capability and is well-aligned with the goals set for a successful LOC device.

## 1. Introduction

The demand for flexible or wearable devices has been increasing rapidly over the past fifty years and there have been many studies addressing the possibilities, applications, and challenges of flexible electronics and integrated systems [1,2,3,4,5]. One of the key applications of the integrated devices is the development of successful and market-ready lab-on-chip (LOC) systems, which aims to bring the whole laboratory process onto a small chip [6,7,8,9,10,11]. LOC devices are miniaturised systems, and the key capabilities of an LOC device include microfluidics (for biosampling purposes) and sensing. These LOC devices aim to manipulate tiny amounts of liquid to achieve various microfluidic functionalities such as sorting, mixing, and transportation of micro-sized droplets. They can also be utilised to sense the composition of the materials fed to the system or to determine their physical specifications. Since the aforementioned functionalities are mostly studied separately and employ different technologies, the practical realisation of an LOC device can be challenging due to the integration and optimisation issues of different devices [12,13,14,15,16,17].

Surface acoustic wave (SAW) devices have been investigated due to their extraordinary capabilities for microfluidic actuations [18,19,20]. SAW actuators are composed of interdigital transducers (IDTs) that are patterned on a piezoelectric substrate, and are capable of generation of acoustic waves that travel along the surface [21,22]. These devices exhibit resonant frequencies, which depend on the phase velocity of the travelling acoustic waves in the substrate and also the period of the IDTs (e.g., f=νλ, in which f is the fundamental resonant frequency, *v* is the phase velocity of the acoustic waves in the substrate, and λ is the wavelength of the IDTs) [21,23]. By applying an RF power to the IDTs at the resonance frequency, SAWs can be generated. When the generated travelling waves encounter a liquid droplet along their path, a portion of their energy will be leaked inside the droplet and this phenomenon can lead to various microfluidic functionalities including streaming/mixing [24,25,26,27,28], separation [29,30,31,32], pumping [22,33,34], jetting [35,36,37,38], and nebulisation [39,40,41] depending on several elements such as the applied power to the IDTs, the surface hydrophobicity, viscosity, and size of the droplet. 

Numerous parameters, e.g., conductivity, mass loading, viscosity, and pressure can affect the phase velocity of the acoustic waves and lead to shifts in the resonant frequencies of these actuators [21]. This mechanism has been successfully utilised for sensing applications. However, achieving higher sensitivity would require the SAW device’s miniaturisation, which needs more stringent controls over cleanroom fabrication processes. On the other hand, electromagnetic metamaterials have been proven to be exceptional wireless sensors with high quality factors. These devices are artificially engineered structures that possess the ability of manipulating electromagnetic waves and exhibit properties such as having negative values of permittivity and permeability simultaneously [42]. Split-ring resonators (SRRs), which are metallic rings with one or more splits fabricated on a dielectric substrate, are among the basic building blocks of electromagnetic metamaterials [43,44,45,46]. Exposing the SRRs to electromagnetic waves can excite resonant frequencies in these structures, and the fundamental magnetic resonance can be modelled as lumped components and will mostly depend on the geometry of the SRRs and the dielectric medium surrounding them (i.e., f=12πLC, in which f is the fundamental magnetic resonance, *L* and *C* are the effective inductance and capacitance, respectively) [47]. Altering any of these parameters leads to a shift in the resonance of SRRs and detecting the shift in the resonance is the key mechanism where SRRs have been employed for biomedical [43,48,49,50], chemical [51,52,53,54], physical [42], and environmental [55,56] sensing applications. Although these electromagnetic metamaterials can be integrated into LOC systems, they cannot make up an LOC device on their own as they lack the capability of microfluidic manipulation. 

Previously, we proposed the idea of combining the concepts of SAW actuators and SRRs utilising a single geometry, and addressed the challenges regarding integration of these two technologies for LOC applications [57,58,59,60,61]. In this study, we aim to explore a new flexible platform for numerous wearable electronic applications. Polymeric substrates are an ideal candidate for creating sensors due to their light-weight, low-cost, and flexibility [62]. They are also compatible with different manufacturing techniques such as standard photolithography and printing methods. However, SAWs have major problems to propagate efficiently on these polymer substrates due to the quick dissipation of the wave energies. 

In this paper, we integrated metamaterials with SAWs, which are directly generated on a piezoelectric thin film coated substrate (silicon wafer as an example in this study) by simply pressing a flexible printed circuit board (FPCB) patterned with IDTs and directly applying resonant frequency signals onto the IDTs. The FPCB manufacturing is a simple, low-cost, and well-matured fabrication technique. We previously demonstrated microfluidic functionalities by pumping a sessile droplet using a similar FPCB pressed onto a LiNbO3 substrate as we reported in Ref. [63]. The originality in this study is that we proposed the IDT structure on the FPCB as an electromagnetic metamaterial-based sensor with three decoupled electromagnetic resonances, which can be explored for different types of physical or chemical sensing applications. The nature of these resonances is explained in the next section. This novel feature is advantageous for sensing applications where distinct resonances can be designed and optimised for different sensing modalities and can be measured simultaneously using external antennas. 

## 2. Sensing Mechanisms Using Flexible Metamaterials

Based on our previous studies reported in Refs. [57,58,59,60,61], it is possible to employ SAW structures as metamaterial-based split-ring resonators. SRRs will have resonant frequencies when excited electromagnetically under various electric and magnetic field configurations (when they are coupled to antennas, e.g., monopole or loop antennas). The nature of the resulting resonance depends on excitation conditions and it can be electric, magnetic, or a combination of both [44,51]. When a circulating current path is induced inside the SRR, the resonance is called a magnetic resonance and the fundamental magnetic resonance can be estimated as lumped components consisting of an effective capacitance and inductance [43]. These components will be affected by the geometry and the dielectric medium of the structure and altering any of these parameters will lead to a shift in the magnetic resonant frequency. Therefore, monitoring the shift in the resonance would be an approach to employ the SRRs as sensors. Moreover, it is possible to induce geometrically different paths of circulating current, thus resulting in different values of effective capacitance and inductance. Obviously, the combination of these values will result in distinct resonant frequencies. If these frequencies can be tailored to be sensitive to separate effects, a single structure can be used to simultaneously measure these effects. 

**Figure 1:**
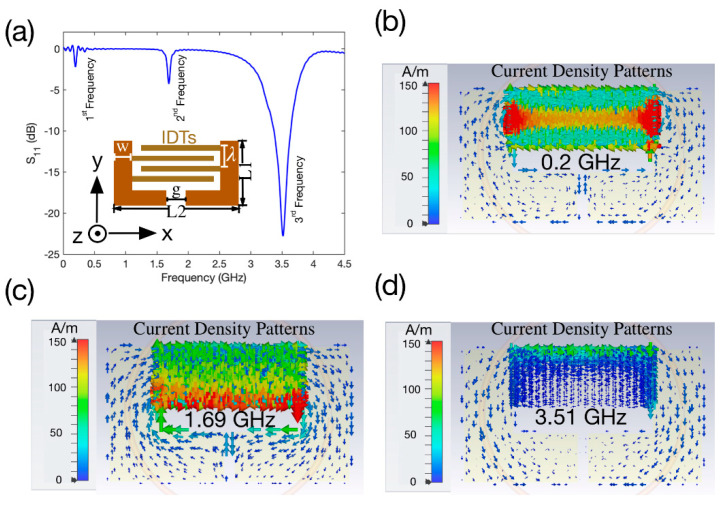
Simulation results of the fabricated device. (**a**) Reflection Coefficient, S_11_, within the range of 0–4.5 GHz. (**b**–**d**) current density patterns at 0.2, 1.69, 3.51 GHz respectively.

Although the resonant behaviour of a basic SRR structure can be estimated using analytic and semi-analytic models, the IDTs of the proposed structure bring complexity that needs to be studied. Therefore, we modelled the structure using an electromagnetic simulator, CST Studio Suite, in a setting where we performed the experiments. The structure located on a substrate with relative permittivity of 3.5 was coupled to a loop antenna with an outer radius of 1.5 cm and an inner radius of 1.4 cm in the simulation environment. An illustration of the studied device is shown in the inset of Figure 1a. The device has the following specifications: number of IDT pairs = 40, length of IDTs—20 mm, *g* = 4 mm, *w* = 7 mm, *L*_1_ = 20 mm, *L*_2_ = 34 mm, λ=200 μm. We then investigated the generated reflection coefficient (S_11_) as well as the current density patterns at the excited resonances. The simulation results are shown in Figure 1a–d. The structure was modelled in 3D using a high frequency module in CST Studio Suite based on the fabricated geometry, and it was excited by plane waves via defined ports along the x-axis. The magnetic and electric fields of the excitation wave were defined along the z-axis and y-axis, respectively, by assigning boundary conditions. Adaptive mesh sizes were used to simulate the structure within the frequency range of interest. 

Figure 1a illustrates the S_11_ scattering parameters of the device within the range of 0–4.5 GHz including the obtained three resonances at 0.2, 1.69, and 3.58 GHz, respectively. The current density patterns at the achieved resonant frequencies are plotted in Figure 1b–d. The current density patterns were plotted by assigning field monitors in the simulator. As can be seen from Figure 1b there are two circulating current paths around the IDTs on the electrodes. Interestingly, the current path does not include the rest of the electrode. The current density is also dominant at the middle of the IDTs with some significance on the edges, which suggests that any effect that results in the relocation of the concentration of the current density can be measured using the first resonant frequency. An example of such an effect is inducing a curvature on the substrate which we investigated experimentally. 

Figure 1c illustrates the current density pattern for the second resonance, in which there is a single loop of circulating current, and it is dominant around the gap region of the structure. The larger current path suggests that the device can be used effectively for material characterisation when it is electrically loaded with samples with different values of permittivity. In addition, the lower edge (with respect to the y-axis) of the IDTs has a higher concentration of current density. Therefore, the second resonant frequency will be more sensitive to droplets placed at this edge.

Figure 1d depicts the current density pattern for the third resonance, in which there are two circulating current loops which are symmetric along the y-axis. Its current density is higher on the upper edge (with respect to the y-axis) of the IDTs. This edge is important for microfluidic functions for which droplets are traditionally manipulated using surface acoustic waves propagating along the y-axis away from the IDTs. Therefore, this frequency can be effectively used to measure droplets placed along this edge. 

## 3. Experimental Methods

### 3.1. Device Fabrication and Characterisation

**Figure 2:**
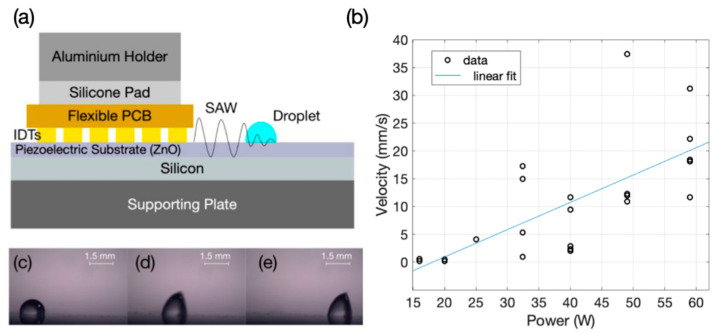
(**a**) An illustration of the experimental setup for microfluidics, (**b**) average pumping velocities achieved by different power levels applied to the electrode pads of the fabricated structure. (**c**–**e**) Snapshots taken of a video with a duration of 3 s at time intervals of 0.42, 0.54, 0.74 s respectively. In this experiment, a 2 µL DI droplet was placed in front of the IDTs and power of 26.2 W was applied at 64 MHz to the electrode pads.

The IDTs made of bilayers of Au/Ni with thicknesses of 30 nm and 2 μm were patterned on a flexible thin polyester laminate (with a thickness of 70 μm) utilising a standard PCB manufacturing process. We used an experimental setting similar to that which we reported in Ref. [63]. A 2-mm aluminium (Al) plate was utilised as the supporting substrate and a 3-inch ZnO coated Si (with ZnO/Si thicknesses of 5/500 μm) wafer was placed on top of the Al plate as the piezoelectric medium needed for SAW generation. In the next step, the fabricated FPCB device was mechanically pressed onto the ZnO/Si wafer from the electrode sides utilising a silicone pad (3 mm thick) and an aluminium holder as illustrated in Figure 2a. 

A vector network analyser (VNA, Keysight E5061B ENA, Santa Rosa, CA, USA) was used to obtain the reflection coefficient, S_11_, of the device. To ensure repeatability, S_11_ was obtained five times by dissembling and reassembling the SAW device and by repeating the characterisation step. The output of a power amplifier (Amplifier research, 75A250, Souderton, PA, USA), connected to a signal generator (Marconi 2024, Plainview, TX, USA) and set at the acoustic Rayleigh frequency, was fed to the electrode pads of the fabricated structure to generate SAWs. The surface of the ZnO/Si was treated hydrophobically by drop coating CYTOP (Asahi Glass Company Ltd., Chiyoda City, Japan) before performing any microfluidic functions. A 2 µL deionized water (DI) droplet was placed in front of the IDTs. The SAW powers at different levels were applied to the electrodes of the fabricated device to transport the droplet. The droplet motion was captured utilising a standard CMOS camera, and the pumping velocity of the droplet was calculated using a video analysis tool (Tracker, Open Source Physics).

### 3.2. Electromagnetic Characterisation and Sensing Experiments

We excited the device wirelessly using a copper loop antenna with a perimeter of 8.8 cm, which was connected to one port of a VNA (Agilent Technologies, N5230A, Santa Clara, CA, USA), and captured the reflection coefficient, S_11_. The device contains three distinct and decoupled resonant frequencies within the range of 0–4 GHz. We verified the sensitive locations of the resonator by placing a 5 µL DI droplet on the gap region, the region on upper IDTs, as well as on both locations at the same time. We monitored the shift in the resonances within the frequency band of interest by capturing the S_11_ signal and explored the possibility of employing each resonance as a separate physical or a chemical sensor.

We utilised the first resonance (0.2 GHz, Figure 2b) as a physical sensor to monitor the flexibility and bending position of the structure. For that, we attached the fabricated device on a flexible mount and placed it on a cylindrical stage. We marked several positions on the right and left sides with equal distances from the cylindrical stage and used the middle point of the cylinder on the substrate as the reference point. We then measured the S_11_ spectrum when the flexible mount was flat on the stage, and also when it was bent and pinned on the marked equal positions on each side of the substrate. We captured the S_11_ responses corresponding to each of the marked locations and monitored the shift in the resonant frequency relative to the case where the flexible mount was flat. 

We utilised the second resonance (1.69 GHz, Figure 2c) as a chemical sensor. To achieve this, we fabricated a micro-droplet holder made of polydimethylsiloxane (PDMS) with the approximate capacity of 40 µL and placed it on the gap region of the resonator. In the first stage of the experiments, we poured 10 µL of DI water and various dielectric solvents sequentially into the PDMS holder and captured the S_11_ spectrum for each one of them. These solvents included methanol, acetone, and isopropanol (IPA). We monitored the shift in the second resonance frequency and compared it with the reference where the holder was empty without any liquid. In the second stage of the experiments, we prepared different volumetric concentrations of mixed IPA and DI. The ratios of the IPA/DI in the prepared solvents were chosen as 4:1 (80% IPA), 2:1 (66% IPA), 1:1 (50% IPA), 1:2 (34% IPA), and 1:4 (20% IPA), respectively. We also included pure IPA (100% IPA) and DI water in the experimental procedure. Next, we poured 20 µL of each concentration of liquid inside the PDMS holder sequentially and measured the S_11_ signals accordingly. We monitored the shift in the resonant frequencies, compared with the reference where the holder was empty without any liquid. 

We utilised the third frequency (3.51 GHz, Figure 2d) as a physical sensor. In the first stage of these experiments, we placed a 5 µL DI droplet on a reference point on the upper IDTs and then translated the droplet upwards and downward on several marked locations on the y-axis. We captured the S_11_ signals corresponding to each of these locations and monitored the shifts in the resonance relative to the case, in which the droplet was absent. In the second stage of the experiments, we placed a DI water droplet of different volumes, ranging from 3 µL to 9 µL, sequentially on the reference point and measured the S_11_ for each case to observe the effects of the droplet size on the shift in the resonant frequency. All the sensing experiments using the metamaterial devices were performed at least five times to ensure reliability. 

## 4. Experimental Results and Discussion

### 4.1. Acoustofluidic Effect

By employing the concept of the SAWs, the flexible PCB device can be utilised to pump a sessile droplet. For this purpose, we adapted the experimental setup which we reported previously in Ref. [63], as explained in the Experimental Section. In our experimental setup, we applied different powers to the electrode on the FPCB, which triggers the SAWs on the ZnO/Si substrate to transport a sessile droplet, which was placed on the ZnO/Si substrate, in front of the pressed FPCB as shown in Figure 2a. When the SAW power is smaller than ~10 W, the streaming phenomena can be observed inside the sessile droplet. Figure 2b illustrates the pumping velocities achieved by applying different power levels to the electrode pads of the fabricated FPCB at 64 MHz for a 2 µL DI droplet. Each data point represents a pumping experiment at a specific input power. The pumping was achievable with a starting power of 16 W and an average velocity of 0.35 mm/s. Further increasing the power led to a generally increasing trend for the pumping velocity with the maximum value of 20.32 mm/s obtained at the applied power level of 59 W. The observed power–velocity relationship within the experimental range is shown with a linear fit in Figure 2b. The variation of velocities at a single power level is large at some power levels due to practical conditions during the experiments, such as maintaining effective contact between the FPCB and the piezoelectric substrate. Figure 2c–e shows the snapshots taken from a 3-s video at 0.42, 0.54, 0.74 s time intervals respectively when the 2 µL DI droplet was pumped at a power of 26.2 W. 

By comparing the obtained results with our previous study reported in Ref. [63], we observed that the achieved velocities were smaller than the ones previously reported on a LiNbO_3_ substrate. This can be attributed to the rougher surface of ZnO in comparison with a LiNbO_3_ surface. Another major reason is that ZnO/Si has a much lower electrocoupling coefficient compared with that of LiNbO_3_ substrate [21]. However, the ZnO/Si substrate is not as brittle as the LiNbO_3_ one, therefore, we could apply a much higher pressure for making and optimising the contact between the device and the substrate. Another advantage is that ZnO/Si can endure much higher powers and can dissipate heat more effectively without damaging the substrate, if compared to LiNbO_3_. 

### 4.2. Sensing Based on Flexible Metamaterials

**Figure 3:**
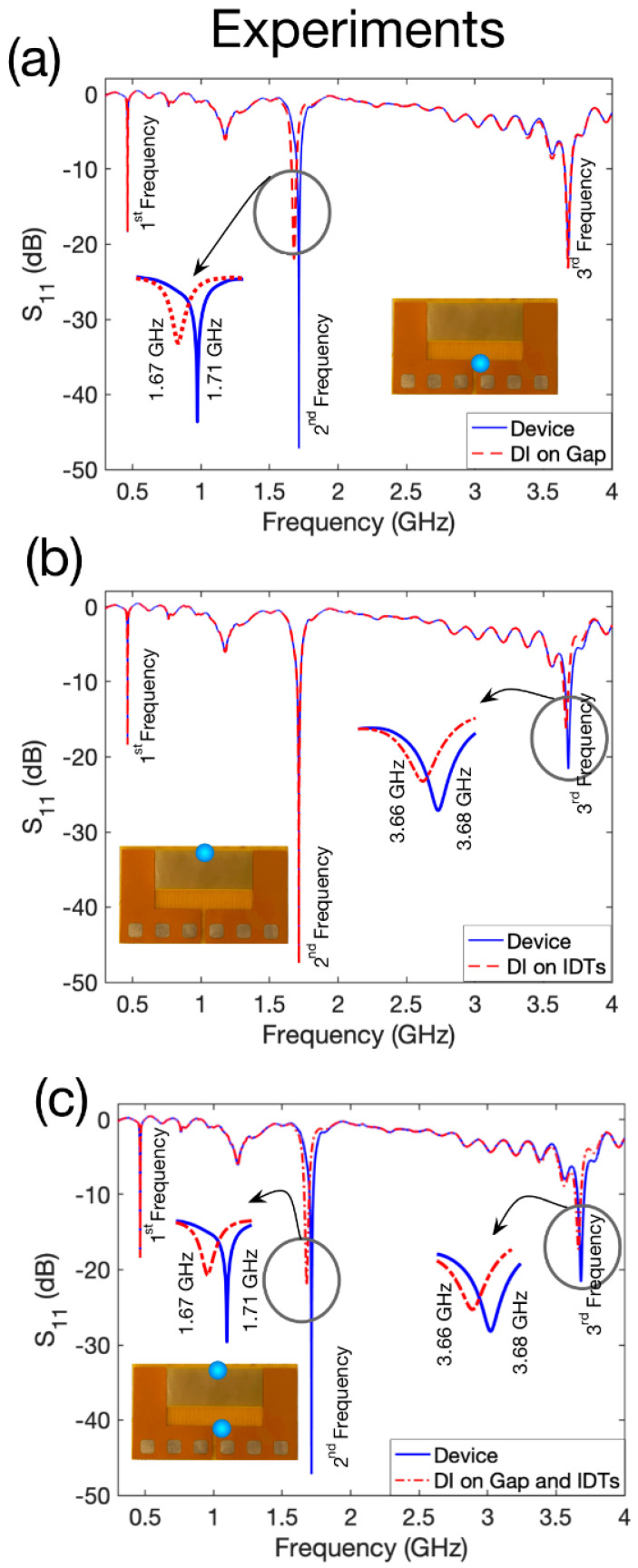
Experimental characterisation of the fabricated device (**a**) when a DI droplet is loaded on the gap as is shown in the inset, (**b**) when the DI droplet is loaded on the IDTs as is shown in the inset, (**c**) when the DI is loaded on the gap and also on the IDTs as is shown in the inset.

We performed experiments by exciting the structure which was placed underneath a copper loop antenna with a perimeter of 8.8 cm. Three resonances can be seen in the S_11_ spectrum shown in Figure 3 at 0.46, 1.71, and 3.68 GHz respectively. The experimental results are in good agreement with the simulation results and the slight discrepancies could be due to the differences in the material properties in the simulator library compared to the actual values in the experiments. In addition, the exact position of the structure relative to the antenna in the simulations and the experiments can have an influence on resonances. 

For the next step of characterisation, we then loaded a 5 µL DI droplet onto the sensitive areas of the second and the third resonant frequencies. We could observe that loading a DI droplet on the gap region of the second structure only affects the second frequency, whereas loading a DI droplet on the upper part of the IDTs has a major influence only on the third resonant frequency. The results of the experiments are shown in Figure 3a–c.

Figure 3a represents the case where the droplet was on the gap region as is shown in the inset of this plot. Since the relative permittivity of the DI has a higher value compared to the vacuum (ε_DI_ = 80, ε_air_ = 1), the expectation is that the effective capacitance would be larger and therefore, the resonant frequency would shift towards lower frequencies [51]. It can be seen that the experiments met our expectations, and the resonance shifted from 1.71 GHz to 1.67 GHz by loading a DI water droplet on the gap. Additionally, we do not see an effect either on the first or the third resonances. On the other hand, we can see that when the DI water droplet is loaded on the upper IDTs as shown in the inset of Figure 3b, only the third resonance shifts towards lower values from 3.68 GHz to 3.66 GHz and there is no obvious effect for the first and the second resonances. Figure 3c indicates the case where the DI droplet is loaded both on the gap and the IDT region, and we can see that both the second and the third resonances shift towards lower frequencies from 1.71 to 167 GHz and from 3.68 to 3.66 GHz, respectively, while there is no effect on the first resonant frequency. To summarise, we showed how the resonances can be decoupled from each other and how each resonant frequency can be employed as a separate sensor, which is advantageous to realise a multiple target sensing device using a single structure.

**Figure 4:**
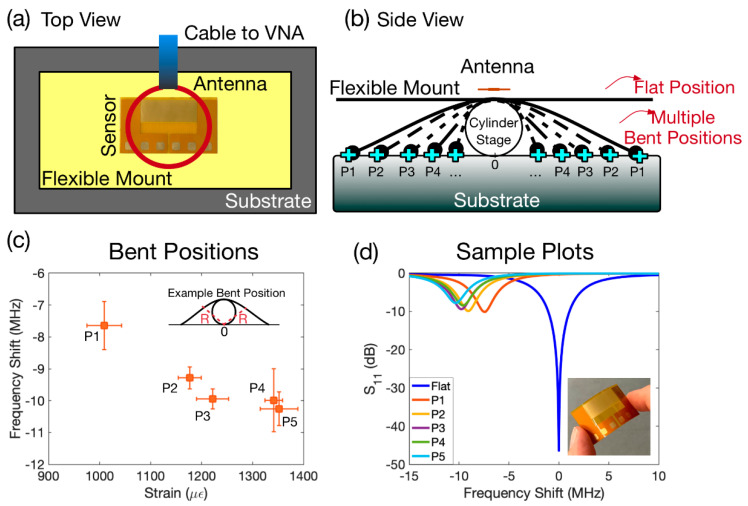
(**a**) Schematic of the experimental setup from top view. (**b**) Schematic of the experimental setup from side view. (**c**) Average nominal strain and frequency shift with respect to the flat position. The radius of curvature as an example of a bent position is illustrated in the inset. (**d**) Sample S_11_ spectra of each position.

In the next step of the experiments, we employed each resonance for a specific sensing application. For the first resonant frequency, we exploited the flexibility of the sensor to monitor the bent position. The experimental setup from top and side views is illustrated in Figure 4a,b, respectively. In this experiment, we attached the sensor on a larger flexible mount (Figure 4a) and placed it on top of a cylindrical stage underneath the antenna as illustrated in the side view of the setup as shown in Figure 4b. We then measured the resonant frequency when the flexible mount was in a flat position, and the reading was 500.93 MHz. For the next step, we bent the flexible mount towards the blue markers, with each marker specified as P_1_ to P_5_ (see Figure 4b), on the substrate and then pinned the structure to that specific position. The distance of the P_n_ on the right and left sides was the same with respect to the centre position of the cylinder on the substrate. Next, we measured the resonant frequency of each bent position starting from P_1_ (when the flexible mount was pinned on P_1_ on both sides) up to P_5_ sequentially. We repeated the experiments for each of the positions at least five times to ensure reliability. We estimated the nominal strain caused by bending using the following formula [64]: ϵ= h/2 R, where ϵ is the nominal strain, h is the thickness of the fabricated substrate, and R is the radius of the curvature (see the inset in Figure 4c). The obtained results are shown in Figure 4c. The points in the plot represent the average values of frequency shift with respect to the flat position (reference point) and the average calculated nominal strain. The error bars represent the standard deviation from the average values. Among the various bent positions, P_1_ and P_5_ showed the minimum and maximum frequency shifts, respectively, compared to the case where the flexible mount was flat. In addition, the minimum and maximum average strains were calculated to be 1009 and 1351 µϵ for P_1_ and P_5_ positions, respectively. 

The loop antenna and the fabricated device under the antenna form a coupled system together and there will an effective capacitance and inductance of the whole system at a resonance, which will in turn determine its resonant frequency. The reason that we see a higher frequency shift in smaller strains versus higher strains could be explained as follows. When the device is flat versus when it is first bent and pinned in the P1 location (lowest strain), there will be a drastic change and deformation of the shape of the device under the antenna. This affects the effective capacitance and inductance and is reflected in the highest change of the resonance. However, when the pinning location changes from P1 to other pinning locations (especially from P4 to P5), the deformation and change of the shape of the fabricated device will not be as drastic as the first case (flat to P1), and therefore, the change in the effective capacitance and inductance will not be as significant. As a result, this will reflect as a lower frequency shift compared to the case where the device is bent from the flat position to the P1 location.

Figure 4d depicts a sample of the calibrated S_11_ spectra for each position relative to the reference point. When the device deviates from the flat position towards the cylinder stage, the frequency shifts towards its lower value. This can be explained by referring to the current density pattern of the first frequency in Figure 1b where the current density is strongest at the edges of the IDT. As the device is bent inwards, this results in an increase in surface capacitance of the device. As a result, the frequency shifts towards lower frequencies.

We employed the second resonant frequency for chemical sensing applications. We utilised various solvents such as methanol, acetone, IPA, and DI water to perform the experiments. We placed in addition a small micro-volume holder made of PDMS on the gap region to precisely control the location of the sample to be loaded on the device and to eliminate any possible effects of the position on the resonant frequency shift. We first measured the resonant frequency when the holder was empty, which was 1.703 GHz. This frequency was the reference point for calculating the frequency shifts. We then loaded 10 µL of each solvent in the PDMS holder and recorded the S_11_ spectrum. We repeated the experiments at least five times for each solvent to ensure reliability. Table 1 lists the relative permittivity values reported [65] for the solvent and their corresponding average resonant frequency (the second resonance) achieved in the experiments. The zero standard deviation reported in Table 1 for the case of air indicates that we measured the same resonant frequency each time the solvents were taken off the device. 

**Figure 5:**
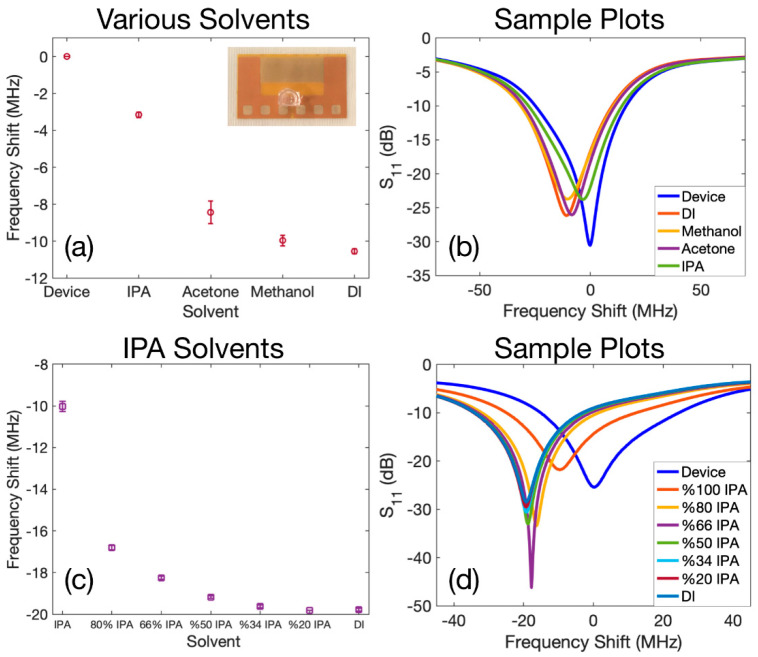
(**a**) Resonant frequencies obtained when various solvents were filled inside the PDMS droplet holder, the Device represents the case where the PDMS holder was empty. (**b**) Sample S_11_ spectra measured for various solvents. (**c**) Average shift in resonant frequencies of prepared IPA/DI solvents compared to the case where the PDMS holder was empty. (**d**) Sample S_11_ spectra for prepared IPA/DI solvents.

Figure 5a shows the average resonant frequency shifts relative to the reference point when a droplet of each solvent was loaded inside the holder, and the error bars indicate the standard deviation from the average value. The “Device” in this plot is the reference point and corresponds to the case where the holder was empty with water. As can be seen from Figure 5a and the values listed in Table 1, solvents with a higher relevant permittivity will increase the effective capacitance of the resonator significantly, leading to a larger shift in the resonance towards lower values. Figure 5b illustrates samples of the calibrated S_11_ spectra for the case when the holder was empty, which is described as Device in the plot, and also shows S_11_ spectra for each of the other solvents.

For the next set of experiments for chemical sensing, we diluted different volumetric proportions of IPA and DI solvents with each other and repeated the sensing procedure. The prepared volumetric IPA to DI ratios were 4:1 (80% IPA), 2:1 (66% IPA), 1:1 (50% IPA), 1:2 (34% IPA), and 1:4 (20% IPA) respectively. We also included results of pure IPA (100% IPA) and DI water in the experimental procedure. By diluting IPA with DI, we varied the effective relative permittivity of the obtained solvent between ε_IPA_ = 18.3 and ε_DI_ = 80. We then loaded the PDMS holder with 20 µL of each prepared solvent. 

The resonant frequency of the device, when the PDMS holder was empty, was measured as 1.7444 GHz. The obtained average frequency shift of each solvent with respect to the case where the PDMS holder was empty is plotted in Figure 5c and the error bars represent the standard deviations. As depicted in this figure, the lowest relative permittivity corresponds to the lowest absolute resonant frequency shift, which is from the pure IPA. By increasing the portion of the DI water in the prepared solvent, the absolute value of the shift in resonant frequency becomes larger. Finally, as expected, the maximum absolute shift of the resonant frequency corresponds to DI water, which has the highest relative permittivity. Figure 5d represents sample calibrated plots of S_11_ spectra for all the prepared solvents. It would be worth highlighting that the volumes of the droplet loaded inside the PDMS chamber in experiments conducted in Figure 5a,b and Figure 5c,d were different from each other. Since the antenna and the resonator would form one coupled system together as mentioned previously, and although the surface area on the resonator is controlled by the PDMS chamber, various sizes of the droplet will still influence the area under the antenna. Therefore, this will affect the effective capacitance of the coupled system of the antenna and the resonator. As a result, the frequency shifts observed for IPA and DI in Figure 5a–d were not the same. 

The set of experiments described for Figure 5 would be ideal for distinguishing various solvents from each other or various volumetric concentrations in one solvent. However, it would be very challenging to combine these two proposed chemical sensing features at the same time. The reason can be attributed to the sensing mechanism of the meta material-based sensors; they can only detect the change in the effective capacitance/inductance and both procedures may have the same effect.

In the next batch of the experiments, we employed the third resonant frequency of the device for physical sensing applications, in which we monitored the position and the size of a droplet. First, the S_11_ characteristics of the device in the absence of a droplet were measured as the starting point (3.6767 GHz). Then, a 5 µL droplet was placed on the location like the inset of Figure 3b and this position was marked as the reference point (0th position) and the S_11_ was measured again in the presence of this droplet. For the next steps, the droplet was moved −7, −3.5, 3.5, 7 mm sequentially along the y-axis relative to the reference point and in each location the reflection coefficient was monitored. This experimental procedure for all locations was repeated at least five times to ensure reliability. 

**Figure 6:**
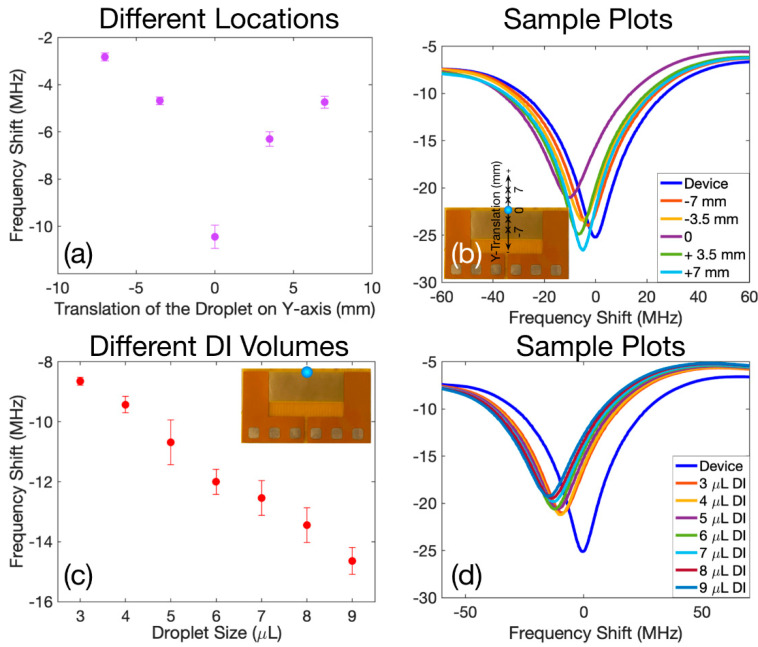
(**a**) The average shift in resonant frequency when a DI droplet was translated along the y-axis relative to a reference point on the upper IDTs and compared to the case, where a droplet is absent. (**b**) Sample S_11_ spectra of the experiments corresponding to transiting DI droplet on y-axis. The inset represents the resonator, the reference point, and the employed locations. (**c**) The average shift in the resonant frequency when a DI droplet with various volumes was placed on the reference point (as illustrated in the inset) relative to the case, where the droplet is absent on the structure. (**d**) Sample S_11_ spectra corresponding to the experiments where the volume of the droplet on the reference point was changed.

Figure 6a,b shows the obtained results. The average shift of the resonant frequency relative to the reference case when there is no droplet on the structure is plotted in Figure 6a and the error bars represent the standard deviation. As depicted in Figure 6a, the maximum absolute shift of the resonant frequency corresponds to the case where the droplet was placed on the reference point, and transportation of the droplet away from the 0th position upwards or downwards decreased the shift in the resonant frequency. 

This phenomenon can be explained by referring to the current density pattern of this specific resonant frequency illustrated in Figure 1d. As can be seen from this plot, the current density is more dominant on the upper IDTs and therefore placing a droplet on this part of the structure will have a more significant impact on the current distribution patterns. As a result, the shift in resonant frequency, in this case, will also be more prominent compared to those at the other regions of the structure. When the droplet is moved away from the sensitive region, both the effective capacitance and inductance decrease, leading to lower shifts in resonance when the droplet is at ±7 mm compared to the case where the droplet is placed at ±3.5 mm. Figure 6b depicts typical examples for the calibrated S_11_ spectra corresponding to all the experimented locations on the device, and these locations are cross-marked on the y-axis in the inset of Figure 6b.

Next, we explored the effects of the droplet volume on the changes of the resonant frequency. For this purpose, we placed a DI water droplet with various volumes starting from 3 µL up to 9 µL sequentially on the reference point as illustrated in Figure 6c. We measured the frequency response in each experiment and calculated the shift in the resonant frequency relative to the reference case where the DI droplet was absent (i.e., 3.6764 GHz). The obtained results are shown in Figure 6c. The points in this plot represent the calculated average shifts in the resonant frequencies and their error bars are the standard deviation. 

As can be seen from this plot, increasing the volume of the DI droplet will cover more of the sensing area of the resonator (where the current distribution becomes denser), and therefore, the effective permittivity of the medium around the IDTs will be larger and as a result, the effective capacitance will be increased. This phenomenon will lead to a larger shift in the resonant frequency. Selected samples of the calibrated S_11_ spectra of the experiments involving different sizes of DI droplets are plotted in Figure 6d. 

The experimental results in Figure 5 and Figure 6 clearly show that for the potential applications of the metamaterial devices for chemical reactions, the exact size and location of the liquid droplets, and the composition of the solvent-under-investigation are needed to be crucially controlled. It also complements the microfluidics functionality, where the droplet can be guided to the area of the interest by applying appropriate SAW power to the electrode pads. 

## 5. Conclusions

In this paper, we reported an integrated platform fabricated on a flexible PCB, which is capable of acoustofluidics based on thin film SAWs and is capable of hybrid sensing (chemical/physical) based on metamaterial-based resonators. The novelty of this work is that the metamaterial device contains three decoupled resonant frequencies for sensing applications and each frequency could be employed as a separate physical or a chemical sensor. We used the first resonance as a physical sensor, in which we bent the device and monitored the shift in the frequency. We used the second resonance as a chemical sensor, by which we detected different dielectric solvents poured inside a micro-droplet PDMS container. We employed the third resonant frequency to detect the size, or the location of a droplet placed around IDTs. The decoupled-resonance feature can be applied to utilise a single geometry for multiple target detections by simultaneously tracking the independent resonant frequencies of the same device. In addition, the resonant frequencies can be processed together to implement a differential readout mechanism to minimise the ambient effects on the measurements.

## Figures and Tables

**Table 1:** Material properties and the mean value of the measured frequencies for solvents.

Material	Relative Permittivity	Resonant Frequency (Mean ± Standard Deviation) (GHz)
Deionised Water	80	1.692306 ± 0.000134164
Methanol	32.6	1.6928862 ± 0.000279697
Acetone	20.6	1.6944072 ± 0.000606935
Isopropanol	18.3	1.69969 ± 0.000151658
Air (device with empty holder)	1	1.702851 ± 0

## Data Availability

Data will be made available upon request.
